# Peripheral blood iNKT cells display an activated profile with both increased apoptosis and dysfunction in obesity

**DOI:** 10.3389/fimmu.2025.1651054

**Published:** 2025-09-10

**Authors:** Chloé Wilkin, Nathalie Esser, Cédric Lassence, Marion Bruneteaux, Marjorie Fadeur, Jenny De Flines, Nicolas Paquot, Jacques Piette, Sylvie Legrand-Poels

**Affiliations:** ^1^ Laboratory of Immunometabolism and Nutrition, GIGA-Immunobiology, ULiège, Liège, Belgium; ^2^ Division of Diabetes, Nutrition and Metabolic Disorders, Department of Medicine, Centre Hospitalier Universitaire (CHU) of Liège, Liège, Belgium; ^3^ Laboratory of Virology and Immunology, GIGA-Immunobiology, ULiège, Liège, Belgium

**Keywords:** iNKT, obesity, lipid antigens, monocytes, activation markers, cytokines, CD1d

## Abstract

Obesity is characterized by a chronic low-grade inflammation and, paradoxically, is also associated with immune cells dysfunction. In this study, we analyzed peripheral blood Invariant Natural killer T cells (iNKT) in individuals with or without obesity. These unconventional T cells recognize lipid antigens presented by the monomorphic CD1d MHC I-like protein. We demonstrated an activation of iNKT cells in individuals with obesity associated with both increased apoptosis and dysfunction as assessed by the lack of responsiveness to PMA/Ionomycin stimulation. This disruption mainly affects the CD4^−^ subset, more dedicated to pro-inflammatory cytokines release and cytotoxicity. Such impact could therefore be involved in the loss of immunosurveillance observed in obesity. Interestingly, CD1d is upregulated on intermediate and non-classical monocytes from individuals with obesity and its expression on both monocyte subsets is correlated with iNKT cell dysfunction. Both the activation and hypo-responsiveness of iNKT cells as well as CD1d modulation on monocytes are significantly reversed after bariatric surgery. Altogether, these data suggest that increased CD1d expression may enhance the presentation of endogenous lipid antigens, thereby contributing to iNKT cell activation in the context of obesity.

## Introduction

Obesity has become a major public health issue, reaching epidemic proportions worldwide. In 2022, more than 2.5 billion adults were classified as being overweight, including 890 million who had obesity (1). Obesity is a chronic multifactorial disease defined by an abnormal and/or excessive accumulation of adipose tissue that can impair health, and predisposes to numerous other non-communicable diseases, such as type 2 diabetes (T2D), cardiovascular diseases and some cancers (2).

A key feature of obesity is the presence of a chronic low-grade inflammation, which plays a key role in the pathogenesis of T2D (3) and the development of cardiovascular diseases and some cancers (4). Paradoxically, obesity has also been linked to impaired immune responses, as evidenced by higher rates of vaccine failure and complications from infection (5) as well as reduced effectiveness of cancer immuno-surveillance and immunotherapy (6). It has become increasingly clear that obesity-associated systemic metabolic modulations contribute to immune dysfunction by disrupting both intrinsic metabolism and functional capacities of immune cells (6, 7). This concept has been illustrated by the work of L. Lynch’s team, which demonstrated the impact of obesity on peripheral Natural Killer (NK) cells (8). Obesity has also been shown to disrupt several populations of human unconventional T cells, like Mucosal-Associated Invariant T (MAIT), γδ T and invariant Natural Killer T (iNKT) cells (9–15).

iNKT cells are a unique subset of T lymphocytes that co-express receptors of the NK lineage along with an invariant T cell receptor (TCR). In mice, invariant NKT TCR combines the canonical Vα14-Jα18 α-chain paired with a limited set of Vβ-chains (Vβ8.1, Vβ8.2, Vβ8.3, Vβ7, or Vβ2). In humans, TCR variability is even more restricted, as this TCR is composed of a Vα24Jα18 α-chain paired with the unique Vβ11 chain (16, 17). Their TCR recognizes lipid antigens presented through CD1d, a non-polymorphic homolog of class I antigen presenting proteins (16). Upon stimulation with lipid antigens, iNKT cells can rapidly trigger effector functions, orchestrating various immune responses against infections, cellular stresses, or tumors (18). New advances support the existence of an ever-increasing repertoire of lipid antigens that are available for CD1d presentation (19). The prototypical antigen for iNKT cells is the α-galactosylceramide (α-GalCer), a glycosphingolipid (GSL) initially extracted from marine sponges (20). Since then, several glycolipid antigens capable of activating iNKT cells have been identified in environmental bacteria, bacteria in the microbiome and pathogens (21). The fact that iNKT cells undergo thymic selection and respond to non-infectious insults implies that endogenous ligands may also control iNKT cell activation. Although several endogenous lipid antigens have been proposed, their antigenicity remains low (22). Interestingly, an α-GalCer variant, with a slightly modified ceramide lipid structure but similar potency to the canonical α-GalCer antigen, was very recently identified in fetal bovine serum, as well as in other mammalian fluids and tissues (23, 24). Due to their thymic development and positive selection by double-positive thymocytes, iNKT cells exit the thymus as functionally mature cells (25). They respond rapidly to antigenic and/or cytokine stimulation by releasing large amounts of Th1, Th2 or Th17 cytokines (26). In addition, upon TCR or NK receptors activation, iNKT cells can also mediate cytotoxicity via perforin and granzymes release (27). The expression of CD4 on human iNKT cells has been used as a predictor of their functional profile, with CD4^+^ iNKT cells being more prone to produce Th2-type cytokines, whereas CD4^−^ (CD8^+^ or double negative) iNKT cells are more associated with pro-inflammatory cytokines production and cytotoxicity (28).

Some studies have reported reduced frequency of peripheral iNKT cells in individuals with obesity, although some discrepancies remain (11–15). Moreover, the phenotype and activity of peripheral iNKT cells in obesity remain poorly characterized and the origin of their decrease is still unknown. In this context, we conducted this study to fully characterize the profile of peripheral blood iNKT cells from individuals with obesity in comparison to lean individuals, and following bariatric-surgery-induced weight loss. Since iNKT cells TCR recognizes lipid antigens presented through CD1d, we also assessed CD1d expression on peripheral blood antigen presenting cells (APCs).

We highlighted, for the first time, an increase in apoptosis of peripheral blood iNKT cells in individuals with obesity, predominantly affecting the CD4^−^ subset. This phenomenon could contribute to the decrease in absolute counts of iNKT cells observed in obesity. Residual iNKT cells in individuals with obesity show an activated, but not exhausted, profile with impaired Th1 cytokines production in response to PMA/Ionomycin stimulation. Interestingly, the activated profile of iNKT cells is correlated with their functional hypo-responsiveness and these disruptions are significantly improved following bariatric surgery-induced weight loss. To further investigate mechanisms underlying peripheral blood iNKT cells activation in obesity, we assessed CD1d expression on peripheral blood APCs. Our results highlighted a significant upregulation of CD1d on both intermediate and non-classical monocyte subsets in individuals with obesity, which was also reversed after bariatric surgery-induced weight loss. These findings suggest that increased CD1d expression may enhance the presentation of endogenous lipid antigens, thereby contributing to iNKT cell activation in the context of obesity.

## Materials and methods

### Study participants

34 individuals with obesity (BMI > 30 kg/m^2^) and 20 lean individuals (BMI < 25 kg/m^2^), age- and sex-matched, were included in this study. Anthropometric, clinical and biological characteristics of the study population are summarized in **Table 1**. Ten lean and ten obese individuals were recruited a second time later for apoptosis analysis on fresh PBMCs. The characteristics of this second cohort are described in **Supplementary Table S1**. For 9 individuals with obesity, blood was harvested before bariatric surgery and on average 9 months afterward (**Table 2**). Blood samples were taken in EDTA tubes after a 12-hour overnight fasting.

**Table 1:** Anthropometric, clinical and biological characteristics of all participants.

Parameters	Overall p-value	Lean	Obese
n (f/m)	NA	20 (14/6)	34 (22/12)
Age (years)	0.4466^#^	42.2 ± 7.9	44.4 ± 11.5
Body Weight (kg)	**<0.0001^#^**	63.9 ± 8.0	107.0 ± 19.3 ***
BMI (kg/m^2^)	**<0.0001^#^**	21.3 ± 1.3	37.6 ± 5.0 ***
Waist (cm)	**<0.0001^#^**	76.2 ± 5.8	114.1 ± 12.1 ***
Fasting glucose (mg/dL)	**0.0443^#^**	89.2 ± 6.5	112.7 ± 58.2 *
Fasting insulin (mU/L)	**<0.0001^#^**	5.24 ± 2.20	14.37 ± 8.61 ***
HOMA-IR	**<0.0001^#^**	1.16 ± 0.51	4.08 ± 3.95 ***
HbA1c (%)	**0.0007^#^**	5.22 ± 0.23	5.96 ± 1.22 ***
Triglycerides (mg/dL)	**<0.0001^#^**	71.3 ± 23.8	167.8 ± 159.4 ***
Total cholesterol (mg/dL)	0.9187^#^	197.3 ± 28.8	204.2 ± 52.9
HDL cholesterol (mg/dL)	**<0.0001^#^**	68.4 ± 10.0	49.7 ± 14.1 ***
Non-HDL cholesterol (mg/dL)	0.0577^#^	128.9 ± 28.8	154.5 ± 52.9
LDL cholesterol (mg/dL)	0.5638	114.7 ± 27.2	119.7 ± 32.8
HDL/total cholesterol	**<0.0001**	0.35 ± 0.07	0.25 ± 0.08 ***
CRP (mg/L)	**<0.0001^#^**	1.74 ± 3.47	9.59 ± 15.15 ***
Type 2 diabetes	NA	0/20	4/34
Metformin treatment	NA	0/20	6/34
Statin treatment	NA	0/20	8/34

BMI, Body mass index; HOMA-IR, Homeostasis model assessment of insulin resistance; HbA1c, glycated hemoglobin; CRP, C-reactive protein; Data are mean ± SD. Unpaired t test or ^#^Mann-Whitney test was performed on data. Significant overall p-value is shown in bold. Lean (n=20) *vs*. Obese (n=34). NA, not applicable.

* p ≤ 0.05; *** p ≤ 0.001.

**Table 2:** Characteristics of the participants before and after bariatric surgery.

Parameters	Overall p-value	Pre-bariatric surgery	Post-bariatric surgery
n (f/m)	NA	9 (8/1)	9 (8/1)
Time since surgery (months)	NA	NA	9.67 ± 4.66
Weight loss (%)	NA	NA	29.6 ± 7.1
Body Weight (kg)	<**0.0001**	104.7 ± 10.3	73.3 ± 6.6 ***
BMI (kg/m^2^)	**0.0039** ^#^	39.8 ± 2.9	27.9 ± 2.8 **
Waist (cm)	**0.0009**	113.6 ± 8.2	89.9 ± 10.2 ***
Fasting glucose (mg/dL)	0.0818	98.1 ± 21.5	85.9 ± 9.6
Fasting insulin (mU/L)	**0.0039** ^#^	13.82 ± 5.52	7.02 ± 4.37 **
HOMA-IR	**0.0073**	3.46 ± 1.79	1.54 ± 1.05 **
HbA1c (%)	0.1184	5.50 ± 0.53	5.29 ± 0.34
Triglycerides (mg/dL)	**0.0195** ^#^	154.4 ± 76.7	94.1 ± 67.7 *
Total cholesterol (mg/dL)	**0.0078** ^#^	191.9 ± 43.9	152.4 ± 16.4 **
HDL cholesterol (mg/dL)	0.5382	53.0 ± 16.1	49.8 ± 7.4
Non-HDL cholesterol (mg/dL)	**0.0030**	138.9 ± 38.2	103.3 ± 20.2 **
LDL cholesterol (mg/dL)	**0.0039** ^#^	107.0 ± 26.0	84.7 ± 18.9 **
HDL/total cholesterol	**0.0274**	0.28 ± 0.08	0.33 ± 0.07 *
CRP (mg/L)	**0.0039** ^#^	10.73 ± 9.31	3.68 ± 4.29 **
Type 2 diabetes	NA	0/9	0/9
Metformin treatment	NA	2/9	2/9
Statin treatment	NA	2/9	2/9

BMI, Body mass index; HOMA-IR, Homeostasis model assessment of insulin resistance; HbA1c, glycated hemoglobin; CRP, C-reactive protein; Data are mean ± SD. Paired t test or ^#^Wilcoxon matched-pairs signed rank test was performed on data. Significant overall p-value are shown in bold. Pre-bariatric surgery (n=9) *vs.* Post-bariatric surgery (n=9). NA, not applicable.

* p ≤ 0.05; ** p ≤ 0.01; *** p ≤ 0.001.

### Reagents and antibodies

Staining was performed using antibodies (**Supplementary Table S2**) from Biolegend, Invitrogen and BD Biosciences. Dead cells were stained with Zombie NIR™ or Zombie Green™ Fixable Viability Kit (Biolegend).

### Preparation of peripheral blood mononuclear cells

PBMCs were isolated from EDTA anticoagulated whole blood from lean individuals and individuals with obesity via Lymphoprep™ Density Gradient Medium (Stemcell™) and SepMate™ tubes (Stemcell™), according to the manufacturer’s instructions. PBMCs were used either directly in flow cytometry analysis or slowly frozen at -80 °C in 90%/10% FBS/DMSO.

### Flow cytometry

**Figure 1:**
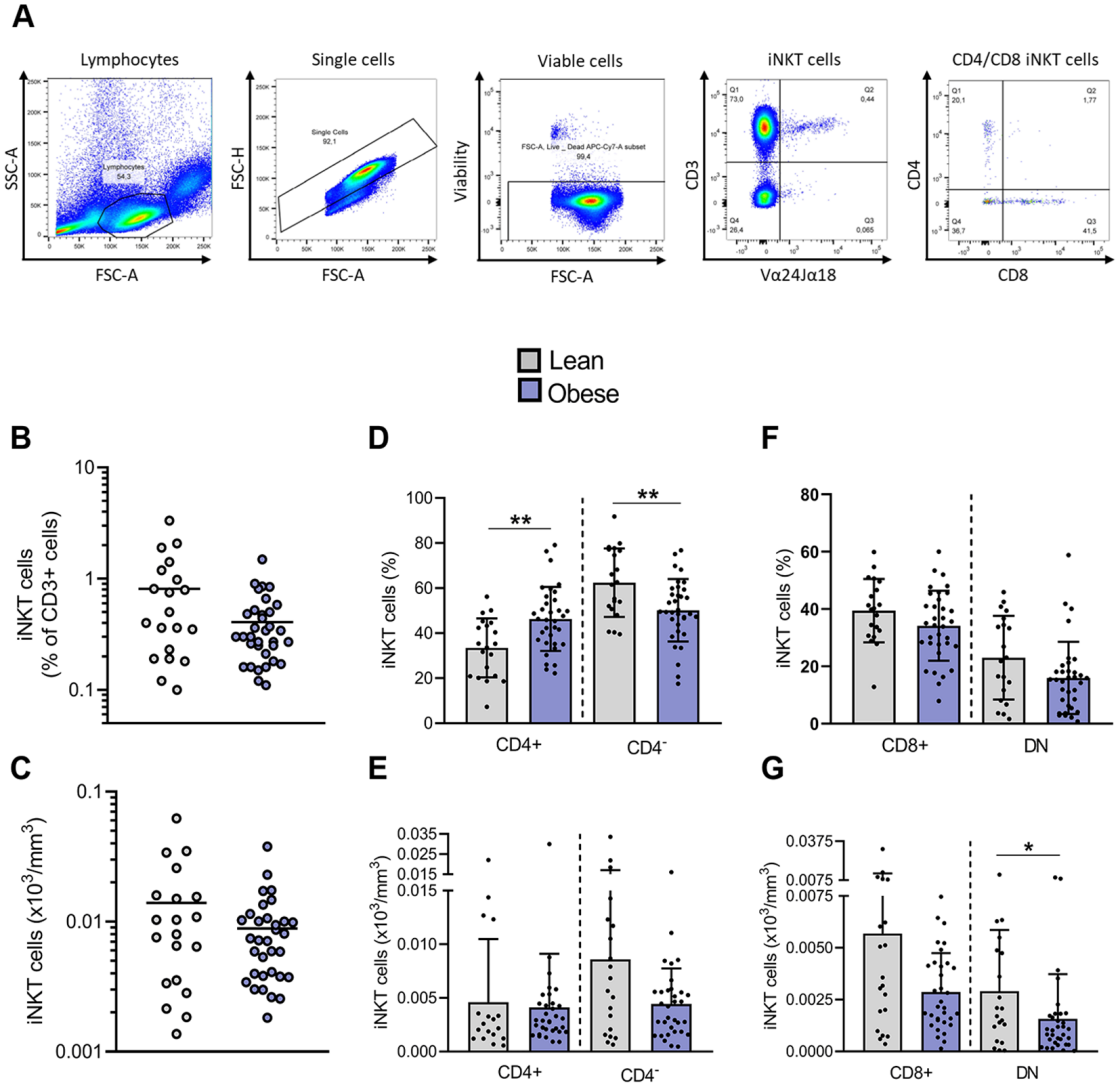
Impact of obesity on frequencies and absolute counts of blood peripheral iNKT cells. **(A)** Representative gating strategy for Vα24Jα18^+^ iNKT cells and their subsets, CD4^+^CD8^−^, CD4^−^CD8^+^ and DN. **(B)** Frequencies of iNKT cells in total CD3^+^ cells. **(C)** Absolute counts of total iNKT cells. **(D)** Frequencies of CD4^+^ and CD4^−^ iNKT cells among total iNKT cells and **(E)** absolute counts of CD4^+^ and CD4^−^ iNKT cells. **(F)** Frequencies of CD4^−^CD8^+^ and DN iNKT cells among CD4^−^ subset and **(G)** absolute counts of CD4^−^CD8^+^ and DN iNKT cells. Lean (n=20) vs. Obese (n=34). Data are mean ± SD. Unpaired t-test or Mann-Whitney test was performed on data according to data distribution. * p ≤ 0.05; ** p ≤ 0.01.

Cells were surface stained with antibodies (see **Supplementary Table S2**) and viability dye (Zombie NIR™ or Zombie Green™ Fixable Viability Kit). For intracellular cytokines profile analysis, cells were stimulated for 4h in RPMI 1640 complete medium containing *PMA/Ionomycin* (Cell Activation Cocktail without Brefeldin A, Biolegend). Brefeldin A (5µg/ml - Biolegend) and Monensin (2µM - Biolegend) were added 1h after stimulation. For intracellular staining, cells were fixed and permeabilized using the Fixation/Permeabilization Kit (Biolegend), according to manufacturer’s instructions. Cells were intracellularly stained with anti-human IFN-γ, IL-4, TNF-α and perforin antibodies (**Supplementary Table S2**). Events were acquired on FACS Verse flow cytometer (BD Biosciences) or ID7000™ Spectral Cell Analyzer (Sony). Analyses were performed with the Flowjo software 10.4 (BD Biosciences). Gating strategies are presented in **Figure 1A** for iNKT cells and **Supplementary Figures S1**, **S2** for monocytes and other APCs, respectively.

### Analysis of iNKT cell apoptosis

Apoptosis was assessed by flow cytometry with fluorochrome labeled inhibitors of caspases (FLICA) from CaspaTag™ Pan-Caspase *In Situ* Assay Kit (Sigma-Merck). Upon isolation, PBMCs were cultured in RPMI 1640 complete medium containing surface antibodies (CD3 and Vα24Jα18, see **Supplementary Table S2**) and FLICA. Cells were cultured under gentle agitation for 1h at 37 °C and 5% CO_2_ atmosphere. Afterwards, cells were washed and fixed according to manufacturer’s instructions. Apoptosis was also assessed by flow cytometry with dual labeling Annexin V-FITC (BD Biosciences) and Propidium iodide (PI – Sigma). Upon isolation, PBMCs were first stained with surface iNKT cell markers (CD3, CD4, CD8 and Vα24Jα18; see **Supplementary Table S2**) before dual labeling with Annexin V and PI according to manufacturer’s instructions. For both apoptosis assays, data were acquired, within 30 min after staining, on CytoFlex (Beckman Coulter) and analyzed with the Flowjo software 10.4 (BD Biosciences).

### Statistics

Data are expressed as mean ± standard deviation (SD). For each readout, normality was assessed using (i) Shapiro-Wilk test, (ii) histogram and (iii) QQ-plot. The unpaired t-test (for normal distribution) or Mann-Whitney test (for non-normal distribution) were performed for two groups comparison (e.g., Lean vs. Obese). The matched-pairs test was used for comparison before and after bariatric surgery-induced weight loss. The tests used for each panel are indicated in the figure captions. All analyses and graphs were performed on GraphPad Prism 10 software. For all analyses, statistical significance was defined as following * p ≤ 0.05; ** p ≤ 0.01; *** p ≤ 0.001. Correlations were performed using linear models (“lm” function). All p-values were adjusted for multiple comparisons using the Benjamini-Hochberg (false discovery rate) method with the “p.adjust” function. Relationships were considered significant when the adjusted-p-value was ≤ 0.05. All correlations analyses were performed using R (version 4.3.2) and the graphs were created using GraphPad Prism 10.

### Study approval

Study was performed in accordance with the ethical principles set forth in the Declaration of Helsinki and received approval from the Ethics Committee of the Liege University Hospital (Ethical committee number: (2022/374). All individuals provided written informed consent.

## Results

### Anthropometric, clinical and biological characteristics of the study population

34 individuals with obesity (BMI > 30 kg/m^2^) and 20 lean individuals (BMI < 25 kg/m^2^), age- and sex-matched, were included in this study (**Table 1**). Similar to the BMI, the body weight and waist circumference of individuals with obesity are significantly higher than in lean individuals. Individuals with obesity are characterized by a significant increase in their fasting insulin, HOMA-IR (homeostasis model assessment of insulin resistance), HbA1c (glycated hemoglobin) and show higher triglyceride levels, lower HDL cholesterol concentrations and HDL/total cholesterol ratio relative to the lean individuals. The pro-inflammatory marker, C-reactive protein (CRP), is also increased in individuals with obesity compared to lean individuals. Four individuals with obesity suffered from T2D, 8 individuals were under glucose lowering treatment (*i.e.*, metformin) and 6 individuals were under cholesterol lowering treatment (*i.e.*, statin).

### The reduction of peripheral blood iNKT cells in obesity mainly affects the CD4^–^ subset

iNKT cells were identified as Vα24Jα18^+^/CD3^+^ cells (**Figure 1A**). We first compared the frequency of peripheral blood iNKT cells between lean individuals and patients with obesity. A trend toward a decreased frequency of iNKT cells among T cells was observed in individuals with obesity, with 0.41% ± 0.29% iNKT cells compared to 0.81% ± 0.83% in lean individuals (**Figure 1B**). However, the absolute count of total iNKT cells was not significantly affected (**Figure 1C**). CD4 expression on human iNKT cells is commonly used as a predictor of functional subsets (**Figure 1A**). CD4^+^ iNKT cells are biased toward Th2-type cytokine production, whereas CD4^−^ iNKT cells, either CD4^−^CD8^+^ or CD4^−^CD8^−^ (DN), are prone to secrete pro-inflammatory cytokines and exert cytotoxic functions (28). Interestingly, obesity significantly disrupted the distribution of iNKT cells between both subsets, leading to a fall in the percentage of CD4^−^ subpopulation in favor to the CD4^+^ subset (**Figure 1D**). The determination of iNKT cells absolute counts clarified the origin of this disturbance: while the number of CD4^+^ iNKT cells remains unaffected, the count of CD4^−^CD8^−^ iNKT cells is significantly decreased in obesity (**Figures 1E–G**).

### Obesity is characterized by an increased apoptosis of peripheral blood CD4^−^ iNKT cells

**Figure 2:**
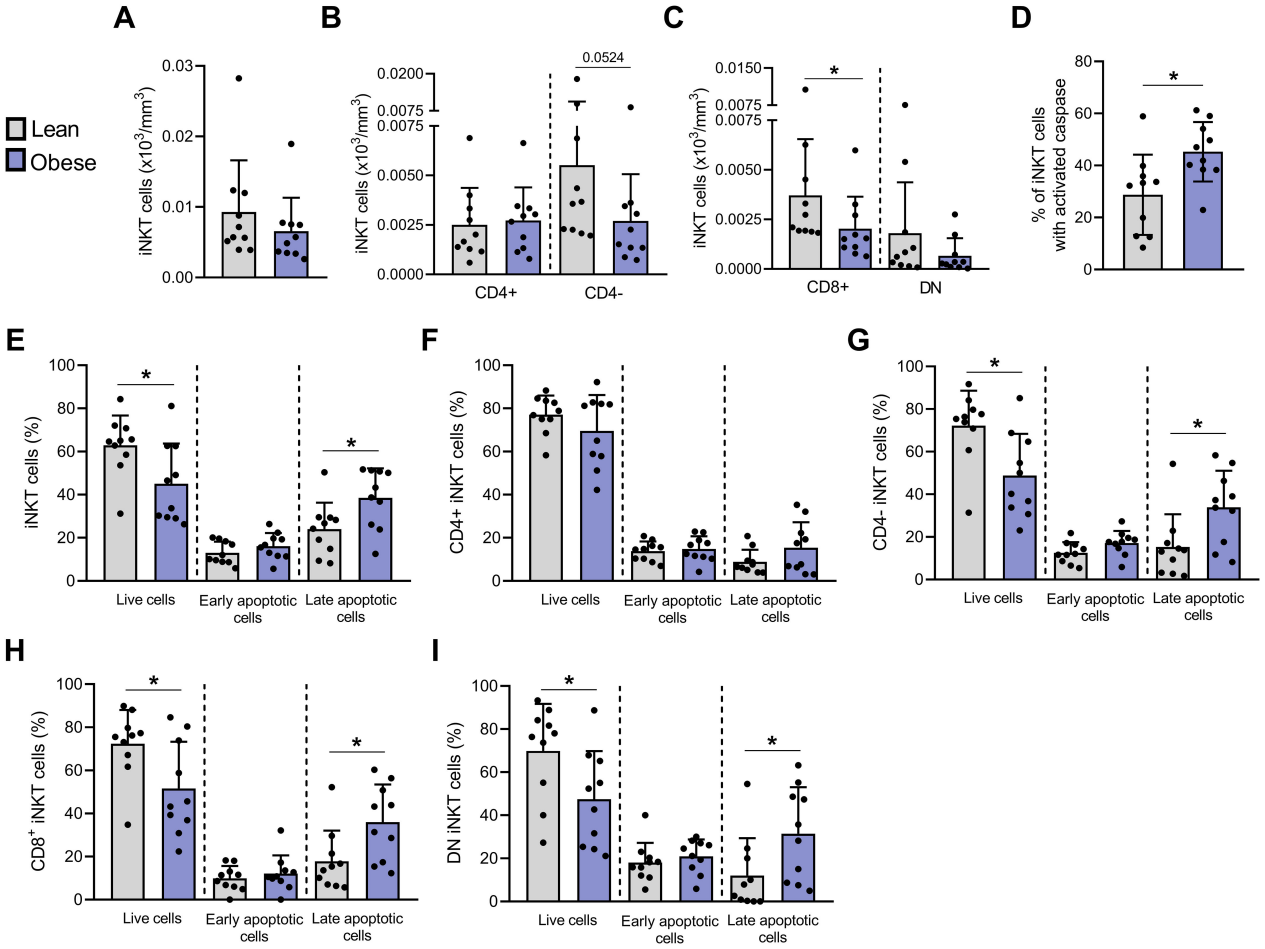
Apoptosis is significantly increased in blood peripheral iNKT cells from individuals with obesity compared to lean individuals. Absolute counts of **(A)** total iNKT cells, **(B)** CD4^+^ and CD4^−^ iNKT cells and **(C)** CD4^−^CD8^+^ and DN iNKT cells. Apoptosis was assessed by flow cytometry with **(D)** FLICA and Annexin V/PI staining on **(E)** total iNKT cells, **(F)** CD4^+^, **(G)** CD4^−^, **(H)** CD4^−^CD8^+^ and **(I)** DN subsets. Lean (n=10) vs. Obese (n=10). Data are mean ± SD. Unpaired t-test or Mann-Whitney test was performed on data according to data distribution. * p ≤ 0.05.

To investigate whether the decrease in peripheral blood iNKT cells observed in individuals with obesity could be associated to an enhanced cell death, we conducted an apoptosis analysis on a subgroup of the larger cohort (n=54), comprising 10 lean and 10 obese individuals. The characteristics of these participants are detailed in **Supplementary Table S1**. These individuals were recruited a second time at a later stage of the study specifically for apoptosis analysis on freshly isolated PBMCs. The absolute counts confirmed the reduction of CD4^−^ iNKT cells in individuals with obesity compared to lean individuals (**Figures 2A–C**). Apoptosis was assessed in freshly isolated PBMCs by flow cytometry using fluorochrome-labeled inhibitors of caspases (FLICA) and Annexin V/PI staining. The percentage of iNKT cells exhibiting active caspases was significantly higher in individuals with obesity (45.27% ± 11.43%) compared to lean individuals (28.74% ± 15.46%) (**Figure 2D**). Similarly, the percentage of iNKT cells in late apoptosis (Annexin V^+^/PI^+^) was increased in individuals with obesity (38.52% ± 13.71%) in comparison to lean individuals (24.00% ± 12.26%) (**Figure 2E**). This increase was predominantly observed in the CD4^−^ population, in both CD4^−^/CD8^+^ and DN subsets (**Figures 2F–I**).

These results demonstrate for the first time an increased apoptosis of peripheral blood iNKT cells in obesity, particularly targeting the CD4^−^ subpopulation, which is consistent with the decrease in absolute counts of this subset (**Figure 2B**).

### Peripheral blood iNKT cells of individuals with obesity display an activated profile

**Figure 3:**
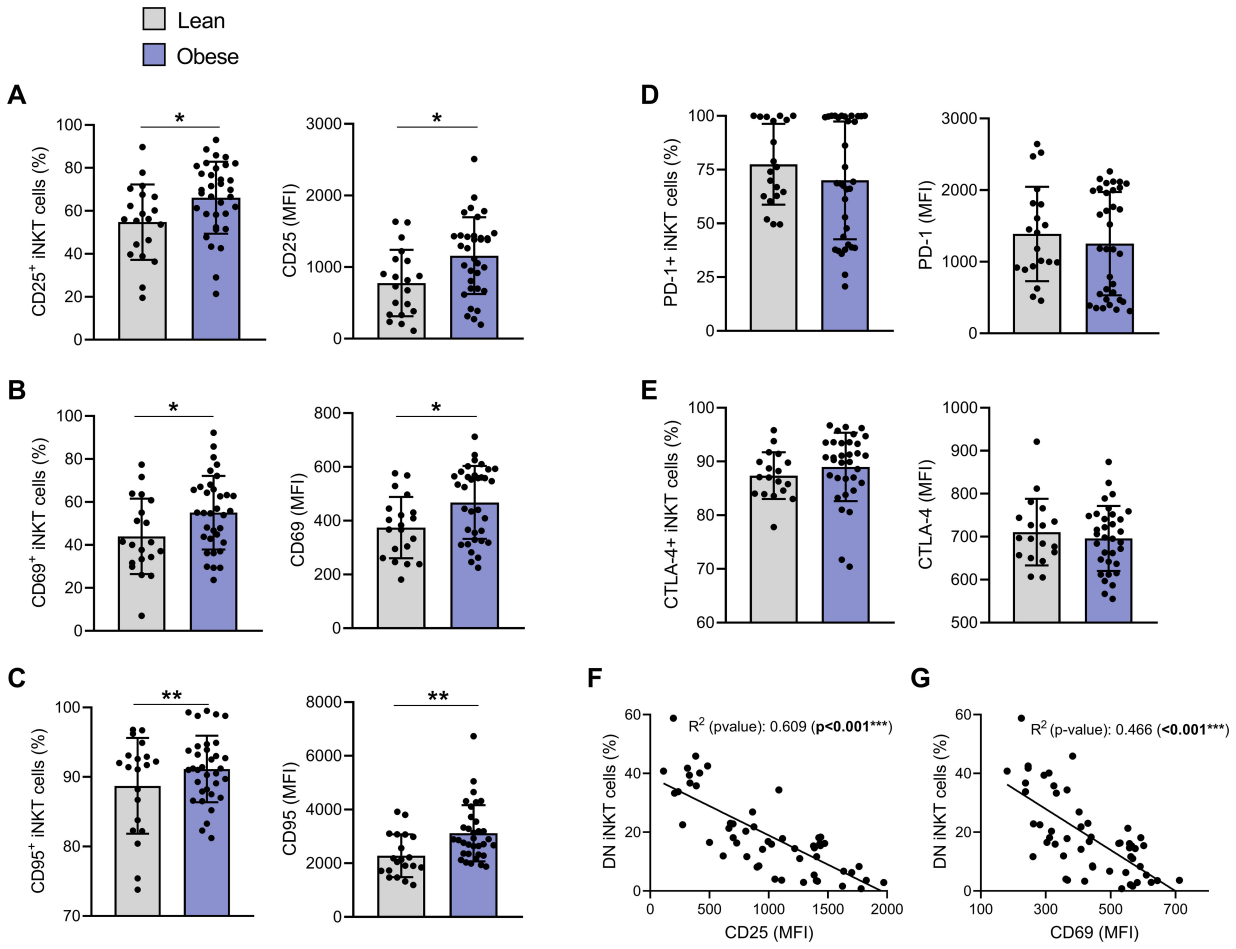
iNKT cells show an activated but not exhausted phenotype in individuals with obesity compared to lean individuals. Percentage of positive iNKT cells and their expression levels (MFI) of: **(A)** CD25, **(B)** CD69, **(C)** CD95, **(D)** PD-1 and **(E)** CTLA-4. Lean (n=20) vs. Obese (n=34). Data are mean ± SD. Unpaired t-test or Mann-Whitney test was performed on data according to data distribution. Pearson correlation of **(F)** CD25 (MFI) or **(G)** CD69 (MFI) and DN iNKT cells frequency (n=54). For correlation, p-values represent significance of associations after correcting for multiple comparisons using the Benjamini-Hochberg method. * p ≤ 0.05; ** p ≤ 0.01; *** p ≤ 0.001.

Since apoptosis of peripheral blood iNKT cells may result from their persistent activation, we next investigated the expression of both early (CD69) and late (CD25) activation markers. CD25 and CD69 were significantly upregulated, both in terms of expression levels (MFI) and percentage of positive cells, with 66.13% ± 16.72% CD25^+^ and 55.01% ± 17.13% CD69^+^ iNKT cells in individuals with obesity compared to 54.77% ± 17.55% and 43.96% ± 17.54% in lean individuals, respectively (**Figures 3A, B**). We also assessed the expression of CD95 (Fas), a cell surface receptor belonging to the tumor necrosis factor receptor superfamily. CD95 has long been recognized as a death receptor mediating apoptosis to maintain immune homeostasis. More recently, non-apoptotic functions have also been attributed to the CD95/CD95L pair, particularly in the regulation of the immune system and T cell activation (29). CD95 (Fas) was also significantly increased (% CD95^+^ cells and MFI) in obesity (**Figure 3C**). In parallel, we assessed the levels of PD-1 and CTLA-4, two markers routinely used to evaluate T cell exhaustion (30). Expression of both exhaustion markers (*i.e.*, percentage and MFI) was relatively high in lean individuals but remained unchanged in individuals with obesity (**Figures 3D, E**). Interestingly, the expression of CD25 (**Figure 3F**), CD69 (**Figure 3G**) and CD95 (data not shown, R^2^ = 0.187, *P* = 0.01) was negatively correlated with the percentage of DN iNKT cells, suggesting that activation of iNKT cells could contribute to their decrease in peripheral blood.

**Figure 4:**
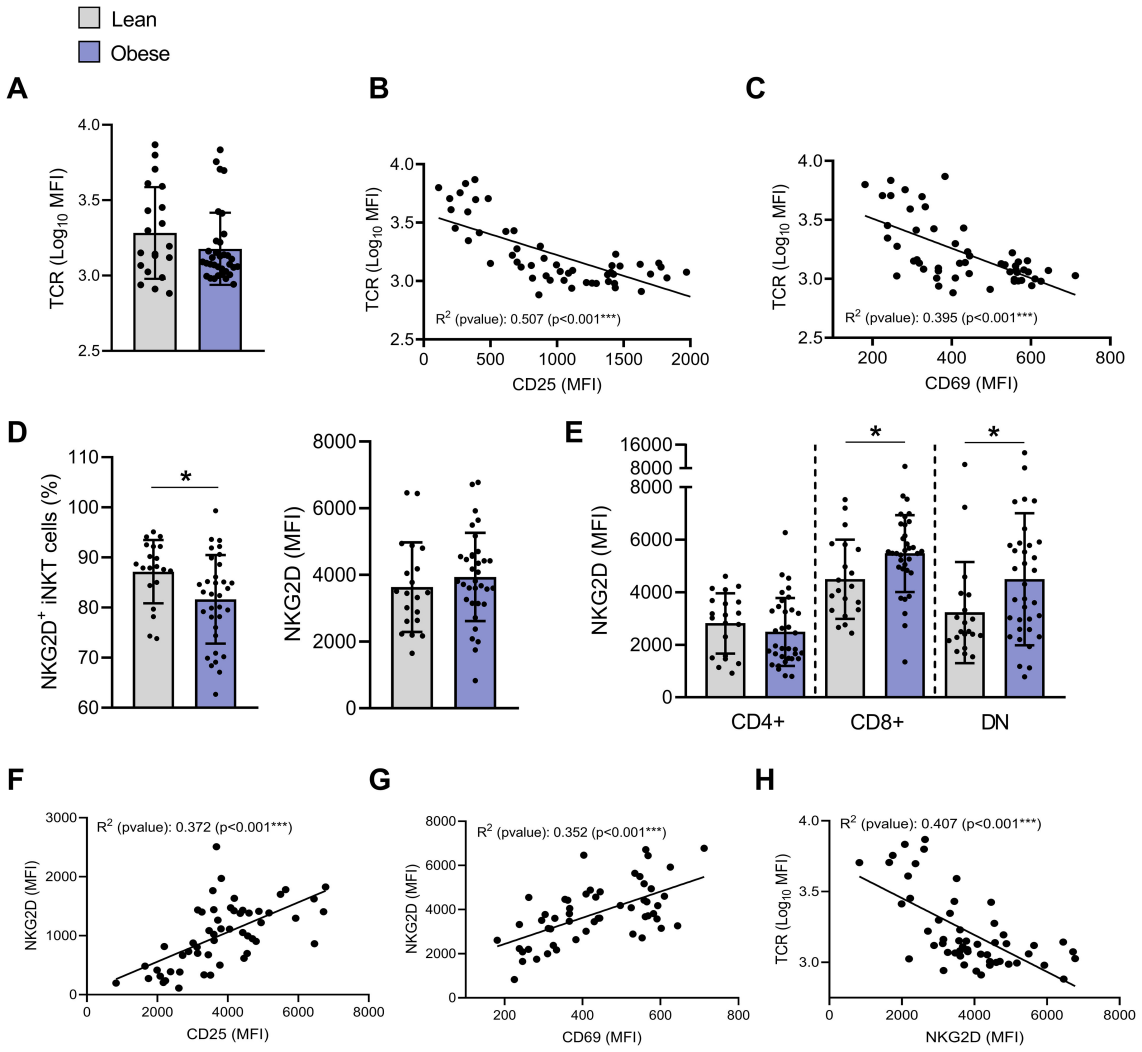
Impact of obesity on TCR and NKG2D expression on blood peripheral iNKT cells. **(A)** TCR (MFI). Pearson correlation of TCR (MFI) and **(B)** CD25 (MFI) or **(C)** CD69 (MFI) (n=54). **(D)** Percentages of NKG2D^+^ iNKT cells and NKG2D expression (MFI) on total iNKT cells. **(E)** NKG2D expression (MFI) on CD4^+^CD8^−^, CD4^−^CD8^+^ and DN iNKT cells in individuals with obesity compared to lean individuals. Lean (n=20) vs. Obese (n=34). Data are mean ± SD. Unpaired t-test or Mann-Whitney test was performed on data according to data distribution. Pearson correlation between NKG2D (MFI) and **(F)** CD25 (MFI), **(G)** CD69 (MFI) or **(H)** TCR (Log_10_ MFI) (n=54). For correlation, all p-values represent significance of associations after correcting for multiple comparisons using the Benjamini-Hochberg method. * p ≤ 0.05; *** p ≤ 0.001.

iNKT cells TCR activation is followed by its internalization (31). Although mean TCR expression level (MFI) was not significantly modulated between individuals with or without obesity (**Figure 4A**), strong negative correlations were observed between TCR levels and both CD25 (**Figures 4B**, R^2^ = 0.507, *p*<0.001) and CD69 (**Figures 4C**, R^2^ = 0.395, *p*<0.001) expression.

We also assessed the expression of the NKG2D, a key receptor that not only drives iNKT cell cytotoxic functions upon ligand engagement, but also amplifies TCR-mediated activation as a co-stimulatory molecule (32). The percentage of NKG2D^+^ iNKT cells significantly decreased from 87.2% ± 6.3% in lean individuals to 81.6% ± 8.8% in individuals with obesity (**Figure 4D**). Since the receptor NKG2D is known to be mainly expressed on CD4^−^ iNKT cells (33), this reduction likely reflects the decrease in CD4^−^ iNKT cells in individuals with obesity (**Figure 1D**). However, overall NKG2D expression (MFI) was not affected by obesity (**Figure 4D**), likely because its upregulation was predominantly observed in CD4^−^ iNKT cell subpopulations, but not in the CD4^+^ subset (**Figure 4E**). As expected (34), NKG2D levels positively correlated with CD25 and CD69 expression (**Figures 4F, G**) and negatively with membrane TCR levels (**Figure 4H**).

**Figure 5:**
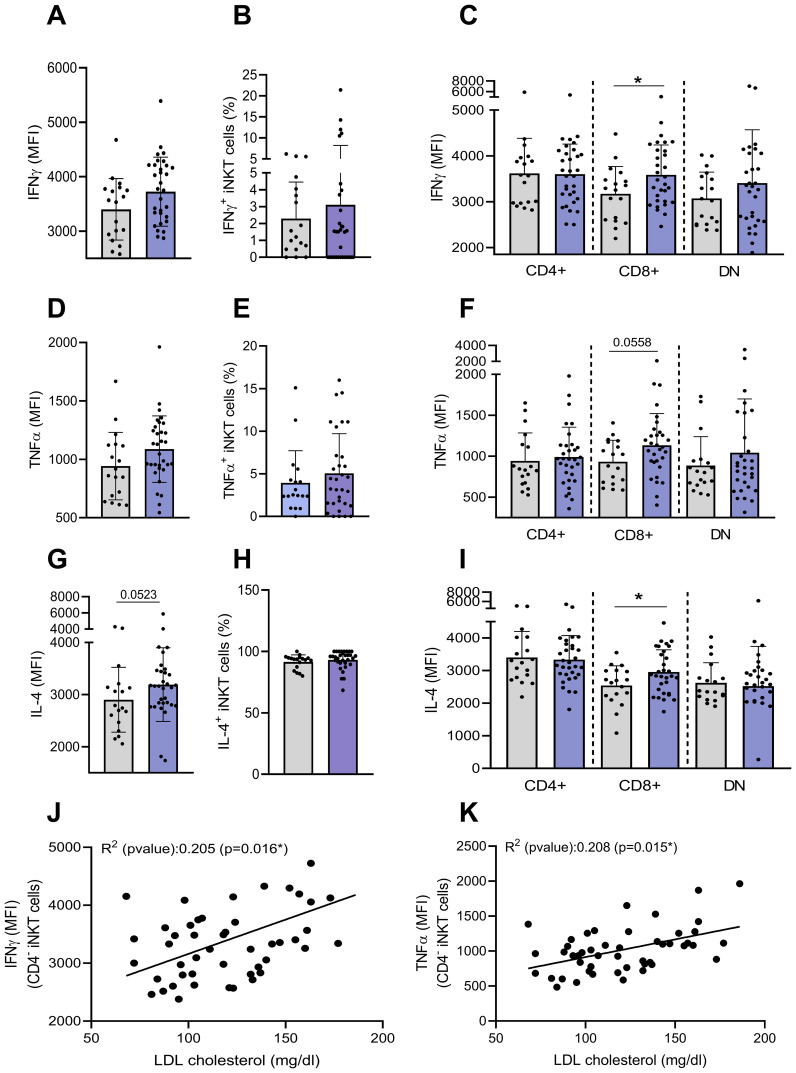
Basal production of cytokines by peripheral blood iNKT cells. The basal intracellular production of IFN-γ, TNF-α and IL-4 was assessed on total and CD4^+^CD8^−^, CD4^−^CD8^+^ and DN iNKT cell subsets by flow cytometry. **(A)** Expression of IFN-γ (MFI), **(B)** percentage of IFN-γ^+^ by total iNKT cells, **(C)** expression of IFN-γ (MFI) by iNKT cells subsets, **(D)** Expression of TNF-α (MFI), **(E)** percentage of TNF-α^+^ by total iNKT cells, **(F)** expression of TNF-α (MFI) by iNKT cells subsets, **(G)** expression of IL-4 (MFI), **(H)** percentage of IL-4^+^ by total iNKT cells, **(I)** expression of IL-4 (MFI) by iNKT cells subsets. Data are mean ± SD. Unpaired t-test or Mann-Whitney test was performed on data according to data distribution. Lean (n=18) vs. Obese (n=32). Correlation between basal production of **(J)** IFN-γ or **(K)** TNF-α by CD4^−^ iNKT cells and LDL cholesterol levels (mg/dl) (n=47). For correlation analysis, all p-values represent significance of associations after correcting for multiple comparisons using the Benjamini-Hochberg method. All analyses were performed on frozen PBMCs.* p ≤ 0.05.

We next determined the basal production of cytokines (IFN-γ, IL-4 and TNF-α) by flow cytometry. A low percentage of iNKT cells constitutively expressed IFN-γ and TNF-α (2.3% and 3.9%, respectively) while nearly all produced IL-4, with no significant difference between groups (**Figures 5A, B, D, E, G, H**). However, CD4^−^CD8^+^ iNKT cells from individuals with obesity exhibited a slight but significant increase in cytokine expression levels (MFI) in comparison to lean individuals (**Figures 5C, F, I**). These findings strengthen previous results suggesting an activation of CD4^−^ iNKT cells in obesity. Interestingly, basal production of both IFN-γ and TNF-α in CD4^−^ iNKT cells seems to be positively correlated with blood lipids levels, including LDL cholesterol (**Figures 5J, K**), triglycerides, total cholesterol, non-HDL cholesterol (data not shown). These correlations should be interpreted with caution given the low R^2^ values. Nevertheless, these correlations of basal cytokine production with blood lipids are the only ones to have a significant p-values since no correlation was observed with other metabolic and inflammatory blood parameters such as fasting insulin, HOMA-IR, fasting glucose, HbA1c or CRP (data not shown).

### Peripheral blood iNKT cells of individuals with obesity display an altered functional profile

**Figure 6:**
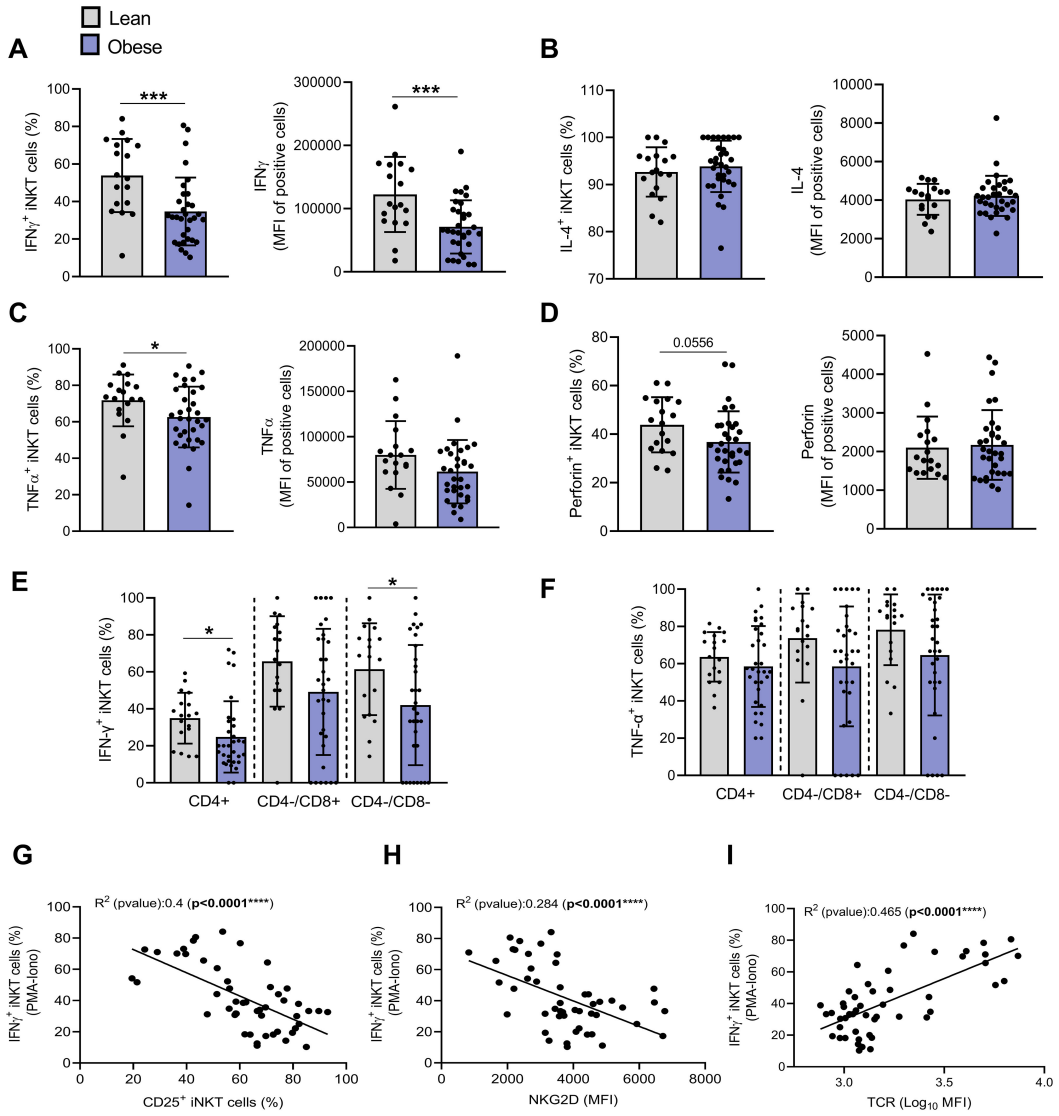
Th1 cytokine production by PMA/Ionomycin-stimulated iNKT cells is impaired in individuals with obesity compared to lean individuals. The production of **(A)** IFN-γ, **(B)** IL-4, **(C)** TNF-α and **(D)** perforin was assessed by flow cytometry in iNKT cells following PMA/Ionomycin treatment (4h) of PBMCs. Intracellular **(E)** IFN-γ and **(F)** TNF-α production were assessed in CD4^+^CD8^−^, CD4^−^CD8^+^ and DN subsets. Data are mean ± SD. Unpaired t-test or Mann-Whitney test was performed on data according to data distribution. Lean (n=18) vs. Obese (n=32). Correlation between percentages of IFN-γ^+^ cells after PMA/ionomycin stimulation and **(G)** the percentage of CD25^+^ iNKT cells, **(H)** NKG2D (MFI) or **(I)** TCR (Log_10_ MFI) (n=47). For correlation analysis, all p-values represent significance of associations after correcting for multiple comparisons using the Benjamini-Hochberg method. All analyses were performed on frozen PBMCs. * p ≤ 0.05; *** p ≤ 0.001; **** p ≤ 0.0001.

We then analyzed the responsiveness of iNKT cells to *ex vivo* stimulation with PMA/Ionomycin. The stimulation-induced production of IFN-γ and TNF-α was significantly lower in individuals with obesity versus lean individuals with 53.88% ± 19.48% and 71.78% ± 14.21% of IFN-γ^+^ and TNF-α^+^ cells in lean individuals versus 34.74% ± 18.08% and 62.62% ± 16.68% in individuals with obesity, respectively (**Figures 6A, C**). A similar reduction was observed for perforin production, although to a lesser extent (**Figure 6D**), whereas no difference was detected between the groups for IL-4 production (**Figure 6B**). Both CD4^+^ and CD4^−^ iNKT subsets exhibited impaired IFN-γ production (**Figure 6E**), while obesity tends to predominantly affect TNF-α production in the CD4^−^ subset (**Figure 6F**). Beyond their reduced frequencies, IFN-γ-positive iNKT cells from individuals with obesity also exhibited diminished cytokine production following PMA/ionomycin stimulation, as demonstrated by the lower MFI of positive cells (**Figure 6A**). Interestingly, iNKT cell responsiveness was negatively correlated with their basal activation level, as shown by inverse associations between IFN-γ production and CD25 (**Figure 6G**), NKG2D (**Figure 6H**), and a positive association with TCR levels (**Figure 6I**).

### CD1d expression is modulated on monocytes subsets from individuals with obesity

**Figure 7:**
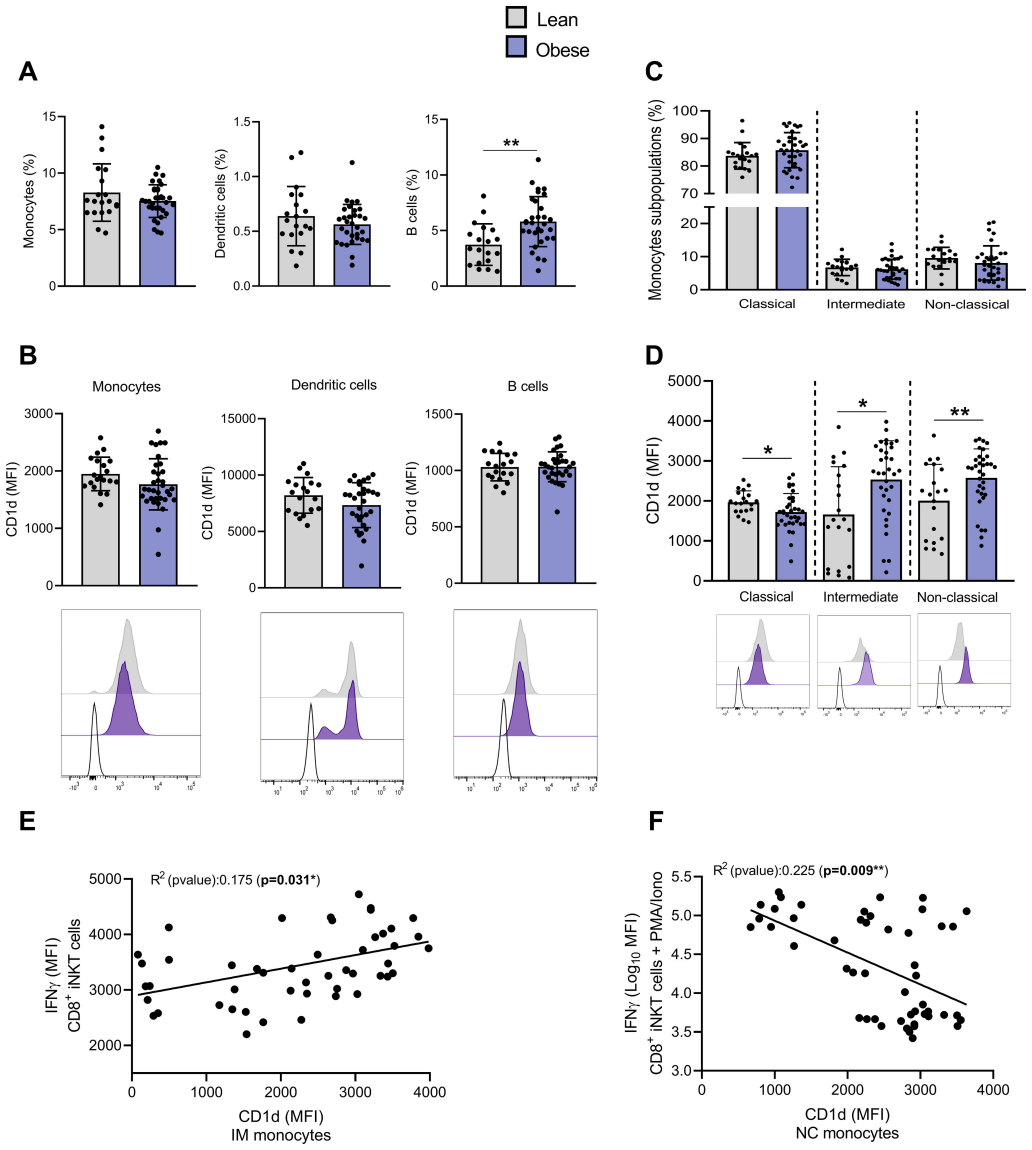
The expression of CD1d is upregulated on intermediate and non-classical monocytes from individuals with obesity compared to lean individuals. **(A)** Percentages of monocytes, dendritic and B cells among PBMCs. **(B)** CD1d expression (MFI) on monocytes, dendritic and B cells. **(C)** Percentages of monocytes subsets (*i.e.*, CM, IM and NCM). **(D)** CD1d expression (MFI) on monocytes subsets (*i.e.*, CM, IM and NCM). Data are mean ± SD. Unpaired t-test or Mann-Whitney test was performed on data according to data distribution. Lean (n=20) vs. Obese (n=34). Analyses on DCs and B-cells were performed on frozen PBMCs (Lean (n=19) vs. Obese (n=31)). **(E)** Correlation between basal production of IFN-γ in CD4^−^CD8^+^ iNKT cells and CD1d expression on IM. **(F)** Correlation between PMA/Ionomycin-induced IFN-γ production in CD4^−^CD8^+^ iNKT cells and CD1d expression on NCM. For correlation analysis (n=47), all p-values represent significance of associations after correcting for multiple comparisons using the Benjamini-Hochberg method. * p ≤ 0.05; ** p ≤ 0.01.

Considering that the activation of iNKT cells can be induced via CD1d-loaded lipid antigen presentation by APCs, we examined CD1d expression on the main peripheral blood APCs. Monocytes and dendritic cells (DCs) frequencies were not affected by obesity, while the frequency of B cells was increased (**Figure 7A**). CD1d expression (MFI) was similar across APC types in individuals with or without obesity (**Figure 7B**). We also analyzed CD1d expression levels on monocyte subsets, *i.e.*, classical (CM, CD14^++^CD16^-^), intermediate (IM, CD14^+^CD16^+^), and nonclassical (NCM, CD14^+^CD16^++^) monocytes. Although monocytes share several common features, each subset has specific functions (35). During bacterial infections, CM are recruited to sites of inflammation, where they recognize and phagocytose pathogens, secrete various proinflammatory cytokines, and recruit other immune cells to regulate the inflammatory response. NCM exhibit distinct motility and crawling pattern along the vasculature. These patrolling monocytes monitor the luminal side of vascular endothelium, where they contribute to vascular homeostasis by recognizing and clearing dying endothelial cells, oxidized low-density lipoprotein (Ox-LDL), and other debris (35, 36). IM express high levels of major histocompatibility complex (MHC) II and are characterized by a high antigen-presenting capacity (35).

No change in CM, IM and NCM frequencies were detected regarding the presence or not of obesity (**Figure 7C**). However, CD1d expression was upregulated on both IM and NCM subsets and downregulated on CM in individuals with obesity (**Figure 7D**). Although we acknowledge the low correlation, the basal IFN-γ (MFI) production by CD4^−^CD8^+^ iNKT cells seems to be positively correlated with the CD1d expression on IM (**Figure 7E**) and NCM (data not shown), while PMA/Ionomycin-induced IFN-γ production appears negatively correlated with the CD1d expression on both NCM (**Figure 7F**) and IM (data not shown). The basal and PMA/Ionomycin-induced TNF-α production showed similar associations with the CD1d levels on monocytes (data not shown).

### Peripheral blood iNKT cell disruption is attenuated after bariatric surgery-induced weight loss

**Figure 8:**
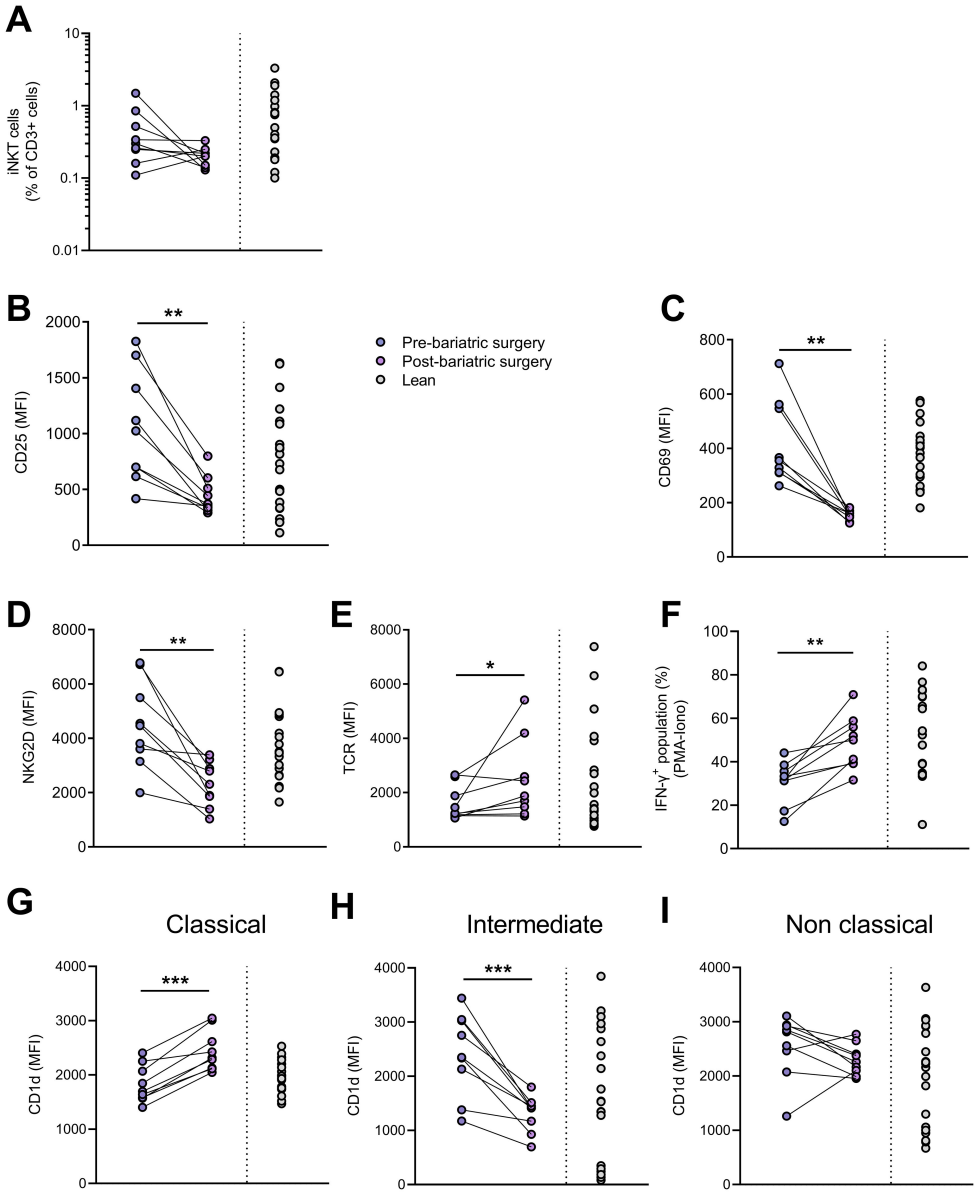
Impact of bariatric surgery-induced weight loss. **(A)** iNKT cell percentages in CD3^+^ population, expression (MFI) of **(B)** CD25, **(C)** CD69, **(D)** NKG2D and **(E)** TCR on iNKT cells, **(F)** percentages of IFN-γ^+^ iNKT cells following PMA/ionomycin stimulation, CD1d expression on **(G)** CM, **(H)** IM and **(I)** NCM in patients before and after bariatric surgery (n=9) and in lean individuals (n=20). Paired t-test or Wilcoxon matched-pairs test was performed according to data distribution. Pre-bariatric surgery (n=9) vs. Post-bariatric surgery (n=9). Analysis of IFN-γ production was performed on frozen PBMCs. * p ≤ 0.05; ** p ≤ 0.01; *** p ≤ 0.001.

Finally, we analyzed peripheral blood iNKT cells in nine individuals with obesity before and nine months after bariatric surgery, a period during which they lost an average of 30% of their weight (**Table 2**). As expected, weight loss led to improvements in lipid profile (LDL-cholesterol, triglycerides,…), insulin sensitivity (HOMA-IR) and systemic inflammation (CRP) (**Table 2**). The frequency (**Figure 8A**) and absolute counts (data not shown) of iNKT cells were not significantly affected post-surgery. However, the activated profile of iNKT cells was markedly attenuated with bariatric surgery-induced weight loss, as shown by the decreased expression of CD25, CD69, NKG2D (**Figures 8B–D**) and CD95 (data not shown) and the increase in the TCR levels (**Figure 8E**). Interestingly, the decreased ability of iNKT cells to respond to PMA/Ionomycin through IFN-γ production was partially restored after weight loss (**Figure 8F**), whereas IL-4 production remained unaffected (data not shown). Concomitantly, the CD1d expression profile on monocyte subsets normalized after weight loss, with upregulation on CM (**Figure 8G**) and downregulation on IM (**Figure 8H**).

## Discussion

Obesity is associated with impaired immune response, as supported by the increased susceptibility to various cancers and a higher risk of complications from infections (5, 6). This immune dysfunction may, in part, results from a negative impact of obesity on immune cell function. In this study, we demonstrated that peripheral blood iNKT cells from individuals with obesity display features of activation, increased apoptosis and functional impairment.

We did not observe a significant decrease in the frequency of iNKT cells among total peripheral blood lymphocytes, but only a trend, in contrast to previous reports (12). This discrepancy could be explained by the significantly higher BMI of the participants included in those studies (> 50 kg/m^2^) compared to ours (> 30 kg/m^2^). Absolute iNKT cell counts are also not significantly different between groups; however, a significant decrease was observed specifically within the CD4^−^ iNKT cell subset, whereas CD4^+^ iNKT cells remained unaffected. Consequently, the distribution of these subsets shifted with an increased proportion of CD4^+^ iNKT cells, at the expense of CD4^−^ iNKT cells, in individuals with obesity. The reduction of CD4^−^ iNKT cells in the peripheral blood of individuals with obesity could result from several mechanisms: increased apoptosis, tissue infiltration, or impaired thymic development. However, studies in high fat diet (HFD)-fed mice reported a decrease in iNKT cell frequencies not only in peripheral blood but also at the level of some tissues (*e.g.*, in adipose tissue, spleen and liver) (12), suggesting that tissue infiltration is unlikely to explain our findings. Moreover, it is well-established that some endogenous glycosphingolipids are critical for iNKT cell thymic development (37). Accordingly, elevated circulating lipid levels could theoretically impair iNKT cell development and reduce their peripheral abundance. However, this hypothesis is challenged by some findings showing that both the numbers and maturation markers of thymic iNKT cells remain largely unaltered in hyper-lipidemic (ApoE-/-) mice (38). Instead, our data support the hypothesis that the iNKT cell defect results from changes in the periphery. Indeed, in our study, we demonstrated for the first time that, in individuals with obesity, peripheral blood iNKT cells, particularly CD4^−^ cells, exhibit increased apoptosis, suggesting that apoptotic death may contribute to their loss. Further, residual iNKT cells from individuals with obesity display an activated profile characterized by increased expression of early (CD69) and late (CD25) activation markers, correlated with the TCR co-receptor, NKG2D. Interestingly, the expression of CD25, CD69 and NKG2D inversely correlate with surface TCR levels, further confirming their activated status. Although we assessed CD25 and CD69 expression across the total iNKT cell population, we have not determined whether activation was uniform across all subsets. Nevertheless, NKG2D expression was significantly and selectively upregulated on CD4^−^ iNKT cells, suggesting that TCR activation in this subset might be reinforced through NKG2D engagement. Basal cytokine production (IFN-γ, TNF-α, IL-4) was also significantly and specifically increased in CD4^−^ (CD8^+^) iNKT cells from individuals with obesity compared to lean individuals. Despite this moderate yet significant basal cytokines production, iNKT cells from individuals with obesity display profound functional defect, characterized by a hypo-responsiveness to *ex vivo* stimulation with PMA/ionomycin, as demonstrated by the marked reduction in Th1 cytokines (IFN-γ, TNF-α) and perforin production. This dysfunction correlates with both increased expression of activation markers and TCR downregulation.

Initially, we hypothesized that iNKT cell activation might lead to exhaustion, characterized by a lack of responsiveness. However, the absence of PD-1 and CTLA-4 upregulation on iNKT cells from individuals with obesity argues against a classical exhaustion phenotype. Previous studies have shown that repeated activation of conventional T cells, such as in chronic viral infections, induces exhaustion (39), while Parekh et al. reported that a single administration of α-GalCer in mice induces a long-term unresponsive anergic iNKT cell phenotype (40). However, this concept of glycolipid-induced anergy was later challenged by Sag et al. (41), who demonstrated that α-GalCer-pretreated iNKT cells actually acquire an active immunoregulatory phenotype characterized by the IL-10 production upon restimulation with α-GalCer, along with a reduced secretion of IFN-γ and IL-4. These IL-10-producing iNKT cells, known as NKT10 cells, represent a distinct regulatory subset, enriched in mouse adipose tissue and also detectable in human PBMCs (42). We did not assess IL-10 production by peripheral blood iNKT cells in our study. However, the phenotype of iNKT cells from C57BL/6 mice intravenously injected with α-GalCer one month earlier appears quite different from that of peripheral blood iNKT cells from obese patients. Notably, these murine iNKT cells show upregulation of CTLA-4 and PD-1 and a downregulation of CD69, CD25 and NKG2D compared to iNKT cells from control mice (41). Further experiments are needed to characterize the phenotype of peripheral blood iNKT cells from patients with obesity, including analyses of proliferation, cytotoxic function, downstream TCR signaling pathways (e.g., Mitogen-Activated Protein Kinase, MAPK) and the expression of anergy-associated genes following α-GalCer stimulation (41). As both activation and exhaustion markers have been defined based on conventional T cells, it is possible that the “dysfunctional” phenotype of peripheral blood iNKT cells from patients with obesity does not align precisely with classical definitions of anergy or exhaustion.

What could drive this activation of peripheral blood iNKT cells in obesity? Direct effects of obesity-related factors (*e.g.*, cytokines, glucose and free fatty acids (FFAs)) or indirect effects via altered self-lipid antigen presentation by CD1d-expressing APCs are both plausible. Lynch’s team previously demonstrated a direct impact of obesity on peripheral blood natural killer (NK) cells involving an intracellular accumulation of lipids impairing NK cells metabolism and therefore their function (8). However, we did not detect increased levels of lipid droplets within iNKT cells of individuals with obesity (data not shown). Furthermore, no correlation was found between blood glucose, FFAs, insulin or CRP levels and iNKT cells parameters. However, BMI correlated positively with CD1d on monocytes (data not shown). Furthermore, blood lipids levels (*i.e.*, total cholesterol, LDL-cholesterol and triglycerides) were associated with basal cytokines production by iNKT cells. In turn, CD1d expression on both NCM and IM correlated with Th1 cytokine levels in iNKT cells, either positively or negatively, depending on whether production was constitutive or induced by PMA/Ionomycin treatment. Based on these preliminary results, we support the hypothesis of an impact of obesity on the presentation of CD1d-loaded lipid antigens by APCs rather than a direct effect on iNKT cells, even if this possibility cannot be excluded.

In peripheral blood, DCs, monocytes and B cells express CD1d and are therefore potential drivers of iNKT cell activation. While no change in CD1d expression was detected on DCs or B cells, a downregulation was observed on CM, whereas IM and NCM showed increased CD1d expression in individuals with obesity. To our knowledge, it is the first study showing CD1d modulation on peripheral monocytes in the obesity context. Indeed, CD1d expression is commonly studied in adipose tissue in the context of obesity. Accordingly, previous studies also revealed an increase in CD1d expression on visceral adipocytes from patients with obesity (43). CM have a very short circulating lifespan (1.0 ± 0.26 days) compared to IM (4.3 ± 0.36 days) and NCM (7.4 ± 0.53 days) (44). Although IM and NCM are much less abundant than CM, their markedly longer lifespan in the bloodstream, their functional characteristics (35, 36), together with CD1d upregulation, could promote their interactions with peripheral blood iNKT cells in obesity.

Like class I and class II MHC, CD1d is constitutively expressed by many cell types and is subject to additional regulation by cytokines (e.g., IFN-γ, TNF-α) or microbial products (45). Interestingly, *CD1D* gene transcription can also be upregulated under sterile conditions. Indeed, activation of the lipid-activated transcription factor, peroxisome proliferator activated receptor gamma (PPARγ), indirectly regulates CD1d expression via the intracellular production of all-trans retinoic acid (ATRA) and activation of the retinoic acid receptor alpha (RARα) (46). Serum lipids such as lysophosphatidic acid and cardiolipin are PPARγ ligands and upregulate CD1d expression on DCs and their ability to activate iNKT cells (47). Oxidized low-density lipoprotein (Ox-LDL) can also elicit retinoid signaling leading to CD1d upregulation (46). Since circulating Ox-LDL levels are increased in obesity (48), they may represent a relevant source of CD1d regulation in this context. Moreover, when whole blood from healthy donors is incubated with various forms of LDL, Ox-LDL is preferentially taken up by CD16^+^ monocytes (49). This is consistent with our results showing CD1d upregulation specifically on IM and NCM. Finally, the correlation observed in this study between LDL cholesterol levels and basal cytokine production by CD4^−^CD8^+^ iNKT cells, although weak, further supports this hypothesis. Nevertheless, this hypothesis remains theoretical and requires further investigation to be confirmed.

The nature and origin of the self-lipid antigen(s) involved remain unclear. These antigens could already be present in the serum of lean individuals but may be more abundantly loaded onto monocytes CD1d in individuals with obesity, thereby promoting iNKT cells activation. Mammalian cells do not synthesize α-GalCer but rather glycosphingolipids (GSLs) with β-linked sugars, which are weak stimulators of iNKT cells. Over the years, several different self-ligands or autoantigens for iNKT cells have been proposed, including phospholipids. Very recently, using supercritical CO_2_ chromatography combined with high-resolution MS/MS, Hosono et al. (24) found α-GalCer compounds (d18:0/16:0) in fetal bovine serum, bovine bile and murine lymphoid organs. These lipids can activate both mouse and human iNKT cell TCR with similar strength to the canonical α-GalCer antigen, despite slight differences in ceramide lipid. Other dihydrosphingosine-based α-GalCer (d18:0) species with various lengths of saturated side chains were also detected in mammals, including in human serum (24). Although antigenic α-GalCer were found in mammalian tissues, their precise origin remains unknown, as no α-specific ceramide galactosyl-transferases have been reported in mammals. Importantly, antigenic α-GalCer detected in this study differ from recently reported antagonistic/weak branched and hydroxyl α-GalCer variants derived from symbiotic bacteria (50, 51). Alternatively, other α-GalCer variants with odd-numbered acyl chains are common in microbes. Accordingly, iNKT cell-activating antigens in the absence of infection could derive from the diet, the microbiome, or others that are truly self. In addition to modulate CD1d expression on peripheral blood monocytes, obesity could also impact the composition and/or levels of these lipid antigens in tissues and serum, contributing to peripheral iNKT cell activation.

In this study, bariatric surgery-induced weight loss and associated improvement in patients’ metabolic and inflammatory status were accompanied by a significant reduction in iNKT cell activation and partial restoration of their responsiveness to PMA/Ionomycin stimulation. The increase in CD1d expression on intermediate monocytes was also reversed. Bariatric surgery significantly decreased circulating lipid levels (triglycerides, total cholesterol, LDL cholesterol) (**Table 2**) and Ox-LDL levels are also known to decrease significantly after bariatric surgery (48). Our hypothesis implicating Ox-LDL in CD1d upregulation on intermediate monocytes and subsequent iNKT cell activation is consistent with our findings after bariatric surgery. These results, however, do not exclude a role for native LDL in facilitating lipid antigen uptake by APCs, as recently described (52). It was surprising to see that the lean individuals’ data looked more like obese individuals pre-bariatric surgery rather than post-bariatric surgery. Indeed, the expression levels of activation markers (i.e., CD25, CD69, NKG2D) appear lower in obese patients after surgery than in lean individuals. This could be explained by the fact that the lean population included individuals with moderate hypercholesterolemia. Since a positive correlation was found between LDL cholesterol and basal Th1 cytokine production by iNKT cells, we can suggest that the expression of activation markers appears lower in obese patients after surgery because they recovered a better LDL cholesterol level (84.7 mg/dL) than in lean individuals (114 mg/dL) (**Tables 1**, **2**).

Obesity is also associated with gut dysbiosis, characterized by reduced microbial diversity and increased abundance of both classically pathogenic species and short-chain fatty acid (SCFA)-producing bacteria (53). The gut microbiome is known to influence iNKT cell biology, potentially through producing glycolipid ligands or microbial metabolites such as SCFAs, amino acids, and bile acids (54). Composition of the gut microbiome is altered in patients after bariatric surgery (55–57). These changes persist beyond the initial weight loss period of 12 months (56). Interestingly, bariatric surgery induces an ‘intermediate’ microbiome phenotype with notably increased microbial diversity but not a complete return to a lean-like gut microbiome composition (57). Thus, if dysbiosis contributes to iNKT cell dysfunction in obesity, it is plausible that this alteration may not be fully reversed, or may even be accentuated, after bariatric surgery.

In conclusion, this study demonstrates for the first time that iNKT cell disruption in obesity is characterized by increased apoptosis and functional impairment, predominantly affecting the CD4^−^ iNKT cell subset. This dysfunction could contribute to the loss of immunosurveillance observed in obesity. Further studies are required to confirm the link between iNKT cell disruption and altered lipid antigen presentation by monocytes, and to elucidate the underlying molecular mechanisms. Such insights could help to identify potential therapeutic strategies targeting iNKT cell dysfunction in obesity.

## Data Availability

The raw data supporting the conclusions of this article will be made available by the authors, without undue reservation.
