# Supplementary material for: Peripheral blood iNKT cells display an activated profile with both increased apoptosis and dysfunction in obesity
Source: Front Immunol. 2025 Sep 10;16:1651054. doi: 10.3389/fimmu.2025.1651054 (PMC12457317; doi:10.3389/fimmu.2025.1651054)
Supplement: Supplementary file 1 [file DataSheet1.pdf]

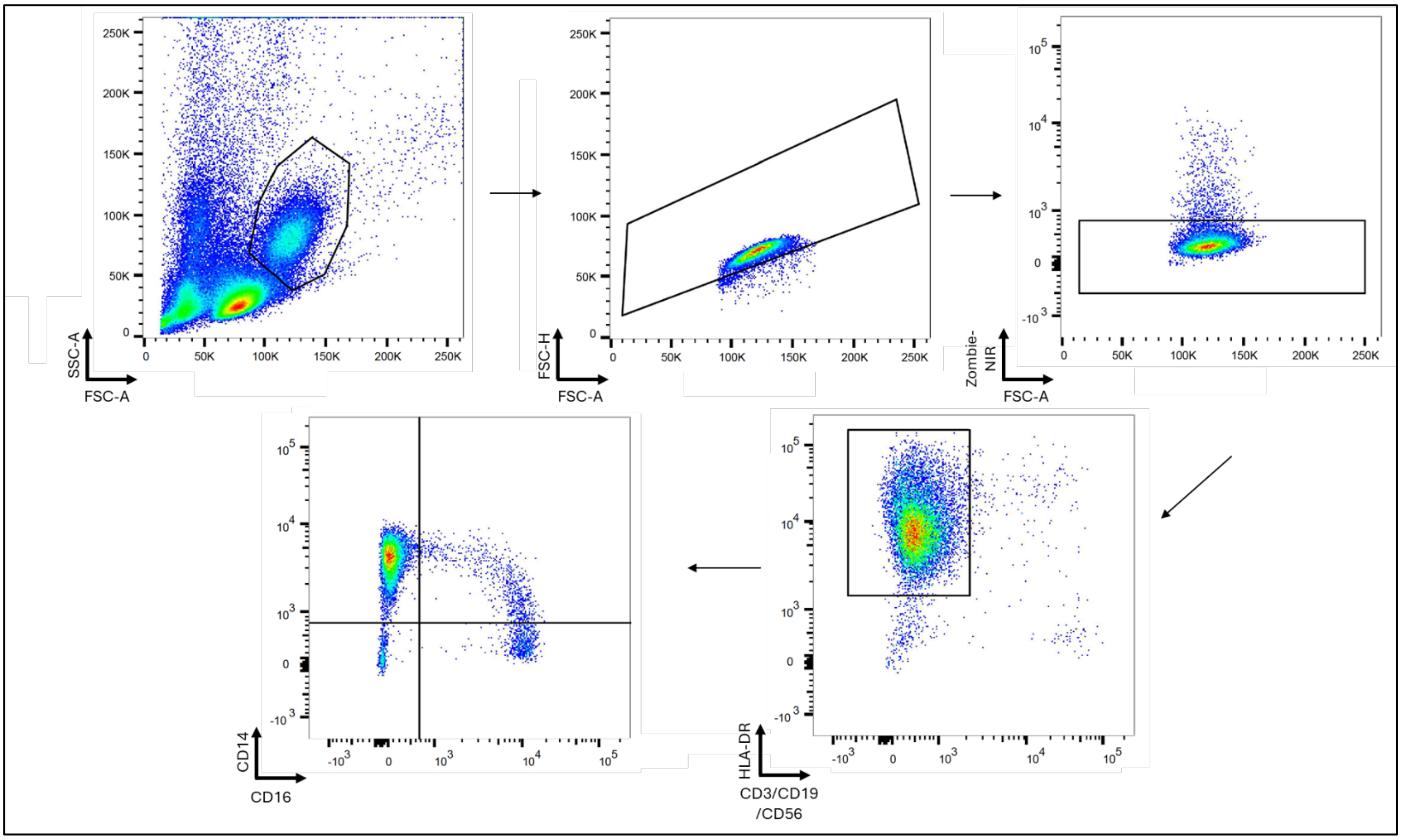

Figure S1. Representative gating strategy for monocytes subsets.

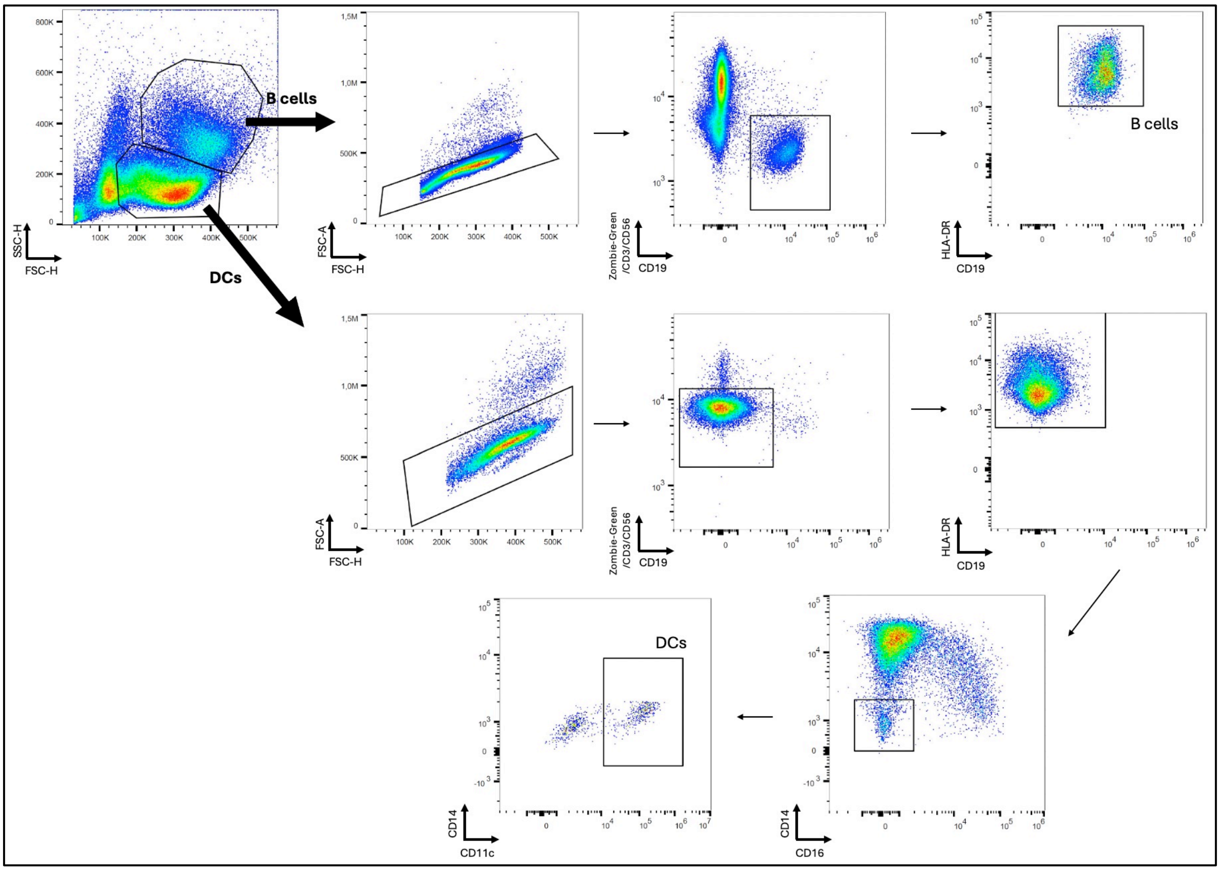

Figure S2. Representative gating strategy for B and DCs.

Table S1. Characteristics of lean and obese individuals for iNKT cells apoptosis assessment

	Overall p-value	Lean	Obese
n (f/m)	NA	10 (7/3)	10 (7/3)
Age (years)	0.6157	47.4 ± 6.5	44.8 ± 14.7
Body Weight (kg)	**<0.0001**[#]	62.4 ± 8.6	97.0 ± 16.1***
BMI (kg/m^2)	**<0.0001**	21.4 ± 1.2	34.1 ± 2.9***
Waist (cm)	**<0.0001**	74.7 ± 5.1	115.9 ± 15.5***
Fasting glucose (mg/dL)	0.5571	86.6 ± 7.1	88.3 ± 5.5
Fasting insulin (mU/L)	**0.0002**	27.60 ± 7.52	64.20 ± 23.20***
HOMA-IR	**0.0002**	0.82 ± 0.32	2.04 ± 0.78***
HbA1c (%)	0.1523	5.16 ± 0.20	5.28 ± 0.16
Triglycerides (mg/dL)	**0.0221**[#]	65.7 ± 16.8	101.1 ± 49.5*
Total cholesterol (mg/dL)	0.7771	194.8 ± 23.1	198.3 ± 30.8
HDL cholesterol (mg/dL)	**0.0039**	68.1 ± 10.1	52.8 ± 10.6**
Non-HDL cholesterol (mg/dL)	0.1630	126.7 ± 20.8	145.5 ± 35.2
LDL cholesterol (mg/dL)	0.2103[#]	113.6 ± 20.5	125.1 ± 31.1
HDL/total cholesterol	**0.0064**[#]	0.35 ± 0.05	0.28 ± 0.09**
CRP (mg/L)	**0.0288**[#]	1.26 ± 0.72	3.10 ± 3.16*
Type 2 diabetes	NA	0/10	0/10
Metformin treatment	NA	0/10	0/10
Statin treatment	NA	0/10	0/10

BMI: Body mass index; HOMA-IR: Homeostasis model assessment of insulin resistance; HbA1c: glycated hemoglobin; CRP: C-reactive protein; Data are mean ± SD. Unpaired t test or [#]Mann-Whitney test was performed on data. Significant overall p-value is shown in bold. Lean (n=10) *vs*. Obese (n=10). NA, not applicable.

Table S2. Anti-human monoclonal antibodies

	Conjugate	Clone	Manufacturer
**CD1d**	APC	51.1	Biolegend
**CD3**	BV510	UCHT1	Biolegend
**CD3**	PE	HIT3a	Biolegend
**CD3**	FITC	OKT3	Biolegend
**CD4**	BV421	A161A1	Biolegend
**CD4**	Spark UV 387	SK3	Biolegend
**CD8**	PerCp	SK1	Biolegend
**CD8**	AF700	SK1	Biolegend
**CD11c**	PE/Cy7	Bu15	Biolegend
**CD14**	eFluor 450	61D3	Invitrogen
**CD14**	PerCP/Cy5.5	HCD14	Biolegend
**CD16**	APC-eFluor 780	CB16	Invitrogen
**CD16**	APC/Fire 750	3G8	Biolegend
**CD19**	PE	4G7	Biolegend
**CD19**	BV785	HIB19	Biolegend
**CD25**	PE/Cy7	BC96	Biolegend
**CD56**	PE	B159	BD Biosciences
**CD56**	FITC	5.1H11	Biolegend
**CD69**	FITC	FN50	Biolegend
**CD95**	PE	DX2	Biolegend
**CTLA-4**	BV785	BNI3	Biolegend
**CXCR6**	BV421	K041E5	Biolegend
**Granzyme**	APC-Fire810	QA16A02	Biolegend
**HLA-DR**	BV510	L243	Biolegend
**IL-4**	PE	MP4-25D2	Biolegend
**INF-γ**	FITC	4S.B3	Biolegend
**NKG2D (CD314)**	FITC	1D11	Biolegend
**PD-1**	BV421	EH12.2H7	Biolegend
**Perforin**	PerCP-Cy5.5	B-D48	Biolegend
**TNF-α**	PE-Dazzle594	MAb11	Biolegend
**Vα24Jα18**	APC	6B11	Biolegend
